# Different Emotional Disturbances in Two Experimental Models of Temporal Lobe Epilepsy in Rats

**DOI:** 10.1371/journal.pone.0038959

**Published:** 2012-06-15

**Authors:** Marion Inostroza, Elena Cid, Liset Menendez de la Prida, Carmen Sandi

**Affiliations:** 1 Instituto Cajal, Spanish National Research Council (CSIC), Madrid, Spain; 2 Departamento de Psicología, Universidad de Chile, Santiago, Chile; 3 Laboratory of Behavioral Genetics, Brain Mind Institute, School of Life Sciences, Ecole Polytechnique Federal de Lausanne (EPFL), Lausanne, Switzerland; Université Pierre et Marie Curie, France

## Abstract

Affective symptoms such as anxiety and depression are frequently observed in patients with epilepsy. The mechanisms of comorbidity of epilepsy and affective disorders, however, remain unclear. Diverse models are traditionally used in epilepsy research, including the *status epilepticus* (SE) model in rats, which are aimed at generating chronic epileptic animals; however, the implications of different SE models and rat strains in emotional behaviors has not been reported. To address this issue, we examined the emotional *sequelae* of two SE models of temporal lobe epilepsy (TLE) – the lithium-pilocarpine (LIP) model and the kainic acid (KA) model – in two different rat strains (Wistar and Sprague-Dawley), which differ significantly in the pattern and extent of TLE-associated brain lesions. We found differences between LIP- and KA-treated animals in tests for depression-like and anxiety-like behaviors, as well as differences in plasma corticosterone levels. Whereas only LIP-treated rats displayed increased motivation to consume saccharin, both SE models led to reduced motivation for social contact, with LIP-treated animals being particularly affected. Evaluation of behavior in the open field test indicated very low levels of anxiety in LIP-treated rats and a mild decrease in KA-treated rats compared to controls. After exposure to a battery of behavioral tests, plasma corticosterone levels were increased only in LIP-treated animals. This hyperactivity in the hypothalamus-pituitary-adrenocortical (HPA) axis was highly correlated with performance in the open field test and the social interaction test, suggesting that comorbidity of epilepsy and emotional behaviors might also be related to other factors such as HPA axis function. Our results indicate that altered emotional behaviors are not inherent to the epileptic condition in experimental TLE; instead, they likely reflect alterations in anxiety levels related to model-dependent dysregulation of the HPA axis.

## Introduction

Affective disorders (e.g., anxiety and depression) frequently co-occur with epilepsy, producing a negative impact on the quality of life of those affected [1], [2]. Although depression and anxiety are common co-morbidities of temporal lobe epilepsy (TLE), the underlying mechanisms are poorly understood. It has been suggested that the affective symptoms associated with epilepsy are secondary to seizures [3], and it has been hypothesized that shared biological factors might both cause seizures and lead to affective disturbances [4].

Several studies have investigated different aspects of affective disorders, including physiological, behavioral and endocrine factors, in experimental models of epilepsy. Using lithium-pilocarpine and kainate *status epilepticus* (SE) models, previous studies reported conflicting results for the performance of epileptic rats in the elevated plus maze, an unconditioned, spontaneous test of anxiety [5]–[7]. In the social recognition test, in which rats are typically exposed to a juvenile conspecific rat, SE models have been shown alterations of socialization, with animals often displaying increased passivity toward the unfamiliar peer [8]. Moreover, it has been reported that animals subjected to lithium-chloride and pilocarpine develop impairments congruent with depression-like symptoms such as anhedonia, which is defined as the inability to experience pleasure from activities formerly found enjoyable (e.g., in rodents, an innate preference toward sweets) [9], along with deregulation of the hypothalamus-pituitary-adrenocortical (HPA) axis [10].

In a recent study [11], we compared two SE models of chronic epilepsy in rats, using systemic administration of lithium-pilocarpine (LIP) or kainic acid (KA). These models are known to reproduce most of the clinical and neuropathological features of human TLE (e.g., hippocampal cell loss, mossy fiber sprouting and dentate gyrus cell dispersion) [12]–[13]. In some of these SE models, neuronal loss is not restricted to the sclerotic hippocampus and occurs in the parahippocampal cortical regions, the thalamus, endopiriform cortex and the amygdala [11], [12], [14]–[17]. At least some of these areas, such as the amygdala, are involved not only in cognitive deficits but also in emotional dysfunctions [18], [19]. The validation of animal models of epilepsy that exhibit co-morbidities with emotional disorders is an important instrument for understanding the underlying mechanisms and for developing new therapies. However, none of the previous studies has compared the impact of different experimental models and rat strain on endocrine factors and emotional behavior.

Here, we examined the emotional behaviors of chronic epileptic rats using two different SE models of TLE (LIP and KA) in two different rat strains (Wistar and Sprague-Dawley). Our previous results indicated that although both models reproduce most features of human TLE, they also differ significantly regarding the pattern and extent of extra-hippocampal brain lesions [11]. Thus, using these models simultaneously allows us to examine the effect of different lesion patterns on emotional performance. Because depression and anxiety in epilepsy have been regarded as multifactorial disorders [20], [21], their investigation should include the identification of different contributing factors, such as the psychological and the endocrine components. Thus, we used different behavioral tasks, including tests for social interaction and anhedonia, to assess depressive symptoms and the open field to evaluate anxiety-like behaviors, and we assessed the activity of the HPA axis by assessing the plasmatic corticosterone level, the major glucocorticoid in rodents.

## Methods

All procedures met the European guidelines for animal experiments (86/609/EEC). Protocols were approved by the Ethics Committee at the Instituto Cajal for the application grants BFU2009-07989 and MemStick (201600).

### Animals

Adult male Wistar and Sprague-Dawley rats weighing 180–200 g were obtained from Harlan Laboratories and our in-house animal facilities at the Cajal Institute in Madrid. Rats were housed in groups of four animals per cage under controlled conditions (22±2°C and 12∶12 light–dark cycle, lights on at 7 a.m.). The animals were given free access to food and water. All procedures met the European guidelines for animal experiments (86/609/EEC).

### Lithium-pilocarpine (LIP) Treatment

Rats from both strains were injected with pilocarpine hydrochloride i.p. 12–24 hrs after receiving an injection of lithium chloride (127 mg/kg, i.p.) [13]. Between one and four doses of 10 mg/kg pilocarpine were injected every 30 min until SE was reached. SE was defined as a condition of continuous seizures lasting longer than 30 min. Diazepam (4 mg/kg, i.p.) was injected 1 hour after SE and repeated during the following 24 hours if convulsive behaviors persisted. Animals were injected with 2.5 ml of 5% dextrose i.p. several times a day, and their diet was supplemented with fruit and powdered milk during the following 2–3 days.

### Kainic Acid (KA) Treatment

Independent sets of rats of both strains were injected with kainic acid (5 mg/kg, i.p.) at hourly intervals until they reached SE [22]. Two to four doses of 5 mg/kg kainic acid were required to reach SE in different animals. Similar to the LIP-treated rats, the convulsive SE was interrupted by diazepam (4 mg/kg, i.p.) one hour after observing the first symptoms. Animals that did not exhibit SE after four doses of 5 mg/kg kainic acid were considered resistant and excluded from the study.

### Control Group

The control group was composed of rats treated with vehicle (saline) instead of pilocarpine or kainic acid in the two different strains, i.e., control Wistar (Control W, n = 13) and control Sprague-Dawley (Control SD, n = 9). Control animals received similar treatment as the experimental group: lithium and diazepam for the lithium-pilocarpine model and diazepam for the kainate model.

### Experimental Design

Epileptic rats were assigned to one of four groups according to treatment and strain: KA-Wistar (n = 8), KA-Sprague-Dawley (n = 7), LIP-Wistar (n = 9) and LIP-Sprague-Dawley (n = 9). Tests typically started approximately 8 weeks post-SE, when animals exhibited spontaneous seizures. Control animals were grouped as outlined above. Rats were handled for 4 days before being subjected to the saccharin reward test to evaluate anhedonia. One week later, animals were evaluated in the open-field test to examine exploration and anxiety-like behaviors and assessed in the social interaction test to detect alterations in social motivation. Possible confounding effects of seizure and other epileptiform activities were carefully controlled using the following measures. First, behavioral tests were suspended for at least two hours for rats that exhibited spontaneous seizures. Second, we considered possible pre-seizure effects by excluding animals that experienced at least one seizure within the hour following test completion. All behavioral tasks were performed between 9:00 and 14:00 hours, except for the anhedonia test, which was performed during the dark phase. We measured the concentration of corticosterone in the plasma twice. The first sample (“pre-behavioral corticosterone”) was obtained before the behavioral testing but after the animal was in the chronic phase of TLE. The second sample (“post-behavioral corticosterone”) was obtained after all behavioral tasks had been performed.

### Anhedonia Test

The examination of anhedonia in rodents takes advantage of their innate preference for sweets and can be carried out using the saccharin consumption test [9]. In this test, when given access to both tap water and a saccharin solution, a healthy subject will strongly prefer the latter, while animals with experimental depression consume equal amounts of water and saccharin. This loss of sweet taste preference has been validated as an indicator of anhedonia [9]. The experimental and control groups were tested at the same time, and all groups received two days of habituation to the new bottle configuration (two bottles filled with tap water placed in the home cage for 12 hours) prior to the test. A two-bottle choice procedure was used to determine baseline saccharin intake. During each test, non-food-deprived rats were presented with two bottles (tap water and 1% saccharin solution) in their home cage for a period of 12 h during the dark cycle. Intake was defined as the difference in bottle weight determined before and after the test. The location of the bottle of saccharin solution relative to the other bottle was counterbalanced across the rats. Saccharin consumption was calculated as the amount consumed in grams per 100 g body weight, and saccharin preference was calculated as saccharin intake/total fluid intake (water + saccharin).

### Open-field Test

Anxiety-like behavior and locomotor activity were assessed in the open-field test, which was conducted within a rectangular arena (50×83×56 cm). The lighting of the arena was adjusted to be 6–8 l× in the center of the maze. For analysis, the floor was divided into two virtual concentric parts, with a central zone in the middle of the arena (30×63 cm) and an exterior area composed of the remaining area along the side walls. At the start of the test, animals were placed in the center of the arena, and their behavior was monitored for 10 min using a video camera mounted on the ceiling above the center of the arena. A computerized tracking system (Ethovision 1.50, Noldus IT, Wageningen, Netherlands) recorded the amount of time spent in each zone and the total locomotion. A measure of innate anxiety level to open field exploration was assessed by estimating the percentage of time spent in the central zone. After each trial, the field was cleaned with acetic acid (0.1%).

### Social Interaction Test

The social interaction test was carried out in a rectangular, three-chambered box (center compartment: 20×50×50 cm; left and right compartments: 40×50×50 cm) fabricated from gray, opaque polycarbonate. The dividing walls had retractable doorways that allowed access to each transparent Plexiglas cylinder. For habituation to the cylinder, each juvenile rat (5–6 weeks old) used in the social interaction test (n = 4) was placed alone in the cylinder for 5 min on 3 consecutive days preceding the social test. The day after the last habituation session, each rat was placed in the center compartment and allowed to explore for 10 min. Next, an unfamiliar juvenile rat was enclosed in one of the cylinders, which was placed in one of the two sides of the social test box during the 5 min session. A plastic bottle was placed in the other cylinder on the other side of the box. Previous pilot studies confirmed that the animals do not show a preference for this object. The locations of the juvenile rat and the object in either the left or right sides of the chamber were counterbalanced between animals. The juvenile unfamiliar rat was constrained within the cylinder to prevent any type of direct physical contact and to ensure that all social approaches were initiated by the test rat. The ratio of juvenile exploration was defined as the time spent exploring the juvenile rat divided by the time spent exploring the juvenile rat plus exploration of the object. The time spent sniffing each cylinder in an active, exploratory behavior toward the juvenile rat or the object was video-recorded and manually scored to evaluate the level of preference for the unfamiliar rat compared with the object. The entire apparatus was cleaned with acetic acid (0.1%) and completely dried between each test.

### Corticosterone Analysis

The concentration of plasma corticosterone was measured twice. “Pre-behavioral corticosterone” values were obtained 8 weeks after SE and prior to the start of the behavioral testing, thus indicating the basal plasma corticosterone of chronic epileptic rats before any stress-inducing behavioral manipulations [23]. The second “post-behavioral corticosterone” sample was obtained one week after completion of all the behavioral experiments. The reasoning behind this measurement was to compare the levels of circulating corticosterone between epileptic and control animals and to examine possible effects of mild stress (behavioral manipulations such as handling and behavioral tests) on the animals. Blood samples were taken by nicking the tail during the light phase (9:00 to 13:00 h), when corticosterone levels have been shown to be low and stable [24]. Blood samples (100 µl) were collected in heparinized test tubes (Sarsted, Nümbrecht, Germany), and the blood was separated by centrifugation for 20 min at 1500 rpm. The plasma was collected and stored at −80°C until the corticosterone levels were assayed by ELISA.

### Statistics

The results are reported as the mean ± SEM unless otherwise indicated. Data analysis was performed using the statistical software SPSS 18.0 for Windows. Mean comparisons were performed with factorial ANOVA, with strain (Wistar and Sprague-Dawley) and treatment (Control, LIP and KA) as the factors. Tukey’s HSD post hoc paired comparison tests were performed when appropriate. The differences between corticosterone samples were analyzed using repeated-measures ANOVA with treatment groups (Control, LIP and KA) including values for both rat strains; for this analysis, groups were pooled because there was no difference between strain and sample time point (pre- or post-behavioral corticosterone) as repeated measures. In addition, we performed Pearson correlation analyses to evaluate possible associations between the emotional behavioral parameters and corticosterone measurements. Results were considered significant at P<0.05.

## Results

We have recently shown that different SE models produce different types of brain lesions and different behavioral outcomes in spatial memory tasks and in unconditioned spontaneous tests for measuring anxiety-like behaviors [11]. More precisely, we examined epileptic rats after either kainic acid or lithium-pilocarpine treatment to induce SE. In this study, using these two SE models, we evaluated whether the emotional behavior of animals was also differentially affected in tests for anxiety- and depressive-like symptoms.

### Depressive–like Behaviors

Depressive symptoms were assessed through the saccharin reward test, which evaluated symptoms of anhedonia (i.e., hyposensitivity to pleasure), and the social interaction test, which compares the willingness to preferentially explore a conspecific rat versus an inanimate object.

**Figure 1:**
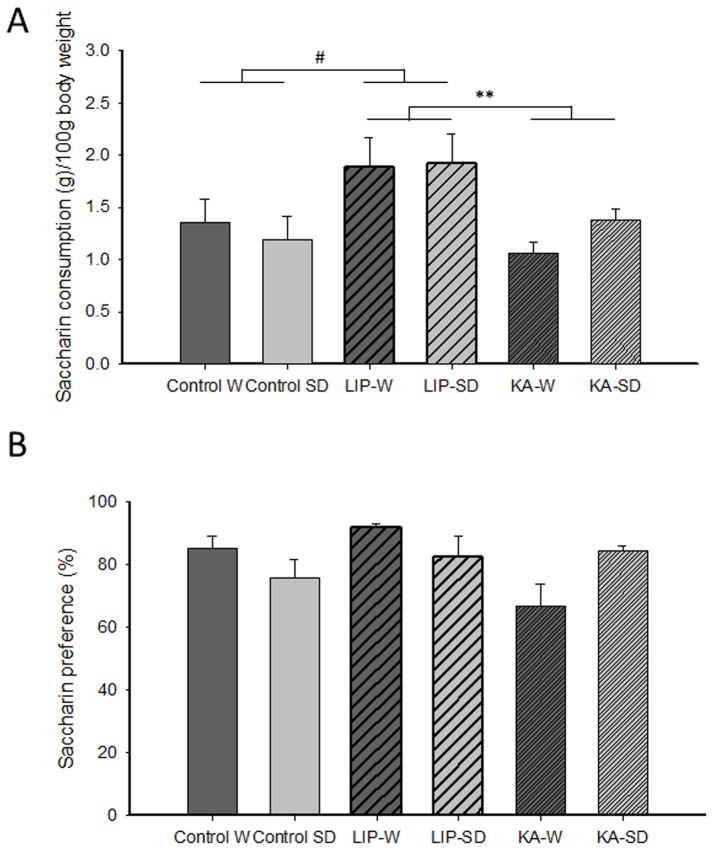
Anhedonia test. (A) Saccharin consumption and (B) saccharin preference was assessed in all groups simultaneously. The data for saccharin consumption per 100 g body weight (A) showed increased saccharin intake in LIP-treated rats compared with controls and KA-treated animals, regardless of the strain. The data for saccharin preference (B) indicated no differences between groups. The results are presented as the mean ± SEM. # P<0.05; ** P<0.01. Abbreviations: W: Wistar; SD: Sprague-Dawley; LIP: lithium-pilocarpine; KA: kainic acid.

A factorial ANOVA on the saccharin consumption in the anhedonia test (Fig. 1A) indicated a lack of effect of strain [F(1, 32) = 0.93, n.s.] or strain x treatment interaction [F(2, 32) = 0.91, n.s.] but a significant effect of the treatment factor [F(2, 32) = 7.43, *P*<0.01]. Further paired factorial ANOVAs including two treatment groups revealed that the treatment effect was mainly due to a significantly increased saccharin intake in the LIP-treated rats compared with the KA-treated rats [F(1, 22) = 19.78, *P*<0.01] and controls [F(1, 18) = 5.03, *P*<0.05]. No differences were observed between KA-treated and control rats [F(1, 24) = 0.75, n.s.]. When saccharin preference was evaluated (Fig. 1B), a factorial ANOVA indicated a lack of effect of strain [F(1, 32) = 3.04, n.s.], treatment [F(2, 32) = 2.57, n.s.] or strain x treatment interaction [F(2, 32) = 0.4, n.s.]. Differences in body weight cannot explain these results because all groups had similar weights at the beginning of behavioral testing (Control W: 430.19±16.58 mg, Control SD: 423.56±12.59 mg, LIP-W: 460.33±27.30 mg, LIP-SD: 420.56±7.21 mg, KA-W: 462.50±14.28 mg, KA-SD: 430.57±10.51 mg, F(2, 49) = 0.56, n.s.). Moreover, LIP-treated rats did not drink more water than the other groups (data not shown). Overall, these results suggest increased motivation in the LIP-treated animals, irrespective of the strain, to consume more saccharin than the other groups.

**Figure 2:**
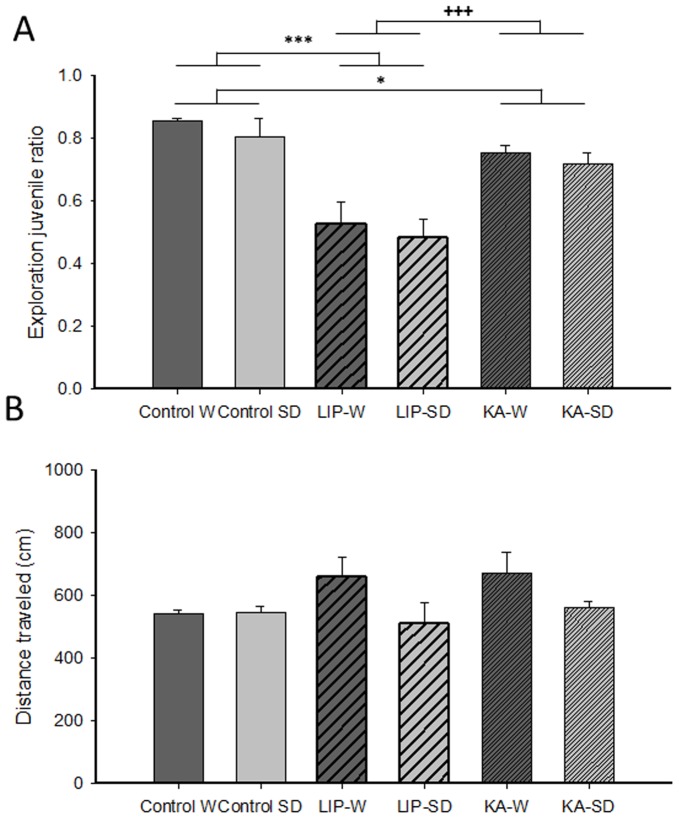
Social interaction test. (A) The juvenile exploration ratio represents time spent exploring the juvenile rat over the total time spent exploring the juvenile rat and the object, evaluated during the 10 min session of the test. Both SE models regardless of the strain, but especially LIP-treated animals, showed decreased preference for exploration of the juvenile conspecific. (B) Total distance traveled in the social interaction test. The results are presented as the mean ± SEM. * P<0.05; *** P<.0001; +++ P<.0001. Abbreviations: W: Wistar; SD: Sprague-Dawley; LIP: lithium-pilocarpine; KA: kainic acid.

Sociability was subsequently assessed using a social interaction test. A factorial ANOVA on the ratio of juvenile *vs*. object exploration indicated a similar lack of effect of strain [F(1, 44) = 1.16, n.s.] or strain x treatment interaction [F(2, 44) = 0.02, n.s.], but a significant effect of the treatment factor [F(2, 44) = 25.04, *P*<0.0001] (Fig. 2A). Further paired factorial ANOVAs including the two treatment groups revealed that this effect was due to both SE-treated groups showing a significantly lower exploration ratio than the controls [LIP: F(1, 27) = 37.82, *P*<0.0001; KA: F(1, 30) = 5.88, *P*<0.05]; additionally, the low exploration ratio of LIP-treated animals was significantly different from that of KA-treated rats [F(1, 27) = 20.01, *P*<0.0001]. These treatment differences in exploration ratio were observed despite a lack of treatment effect in total locomotor behavior [F(2, 44) = 1.36, n.s.](Fig. 2B). Locomotion data, however, depicted an effect of strain [F(1, 44) = 5.33, *P*<0.05], reflecting a mild increase in distance moved by Wistar SE-rats. No strain x treatment interaction was found for locomotor behavior in this test [F(2, 44) = 1.73, n.s.]. Overall, these results indicate reduced motivation for social contact in both SE models irrespective of the strain, with LIP-treated animals being particularly affected.

### Anxiety-like Behaviors

**Figure 3:**
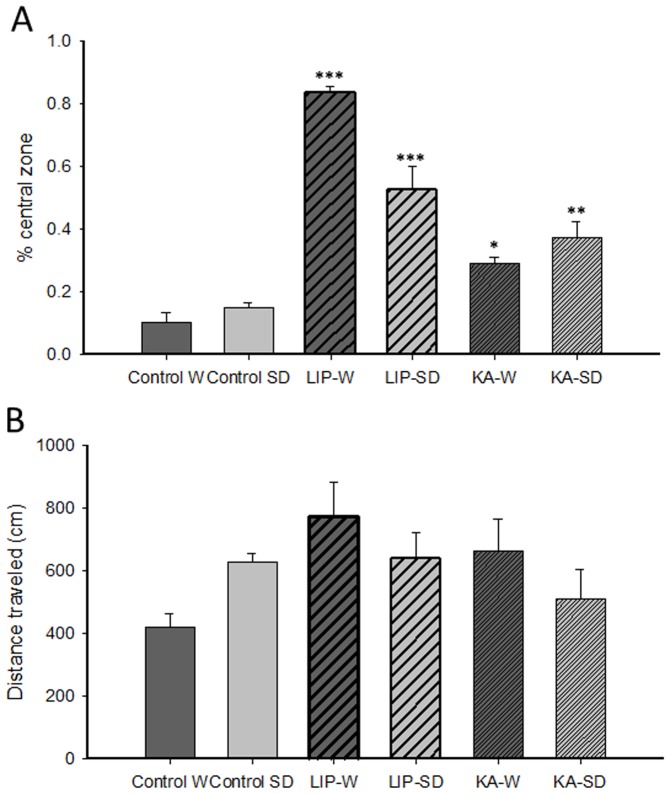
Open field test. (A) Exploration of the central zone was measured as an index of anxiety-like behavior. LIP- and KA-treated rats from both strains showed increased exploration of the central zone. (B) Total distance traveled. The results are presented as the mean ± SEM. * P<0.05, ** P<0.01, and *** P<0.0001 vs. their respective control groups. Abbreviations: W: Wistar; SD: Sprague-Dawley; LIP: lithium-pilocarpine; KA: kainic acid.

Anxiety-like behaviors were assessed in the open field by evaluating the time spent in the central zone and total distance traveled. A factorial ANOVA on the percentage of time spent in the central zone (Fig. 3A) indicated a significant effect of the treatment [F(2, 40) = 70.39, *P*<0.0001] and the strain x treatment interaction [F(2, 40) = 10.57, *P*<0.0001], but a lack of effect of strain [F(1, 40) = 2.49, n.s.]. The treatment effect was due to the increased time spent in the central zone of the arena by both treated groups compared to controls [LIP: F(1, 26) = 115.57, *P*<0.0001; KA: F(1, 28) = 47.85, *P*<0.0001] and to the higher time spent in this zone by the LIP-treated animals versus KA-treated rats [F(1, 26) = 39.68, *P*<0.0001]. Post hoc analyses following the interaction of the 2×3 factorial ANOVA further revealed that the percentage of time spent in the central area was significantly higher for LIP-treated rats than for controls in both Wistar (*P*<0.0001) and Sprague-Dawley strains (*P*<0.0001). The same pattern was found in KA-treated animals when compared with their control group (Wistar: *P*<0.05; Sprague-Dawley: *P*<0.01). Furthermore, a factorial ANOVA performed on the distance traveled in the arena (Fig. 3B) failed to indicate an effect of strain [F(1, 40) = 0.15, n.s.] or a strain x treatment interaction [F(2, 40) = 3.33, n.s.] but revealed a tendency towards statistical significance for the treatment factor [F(2, 40) = 3. 51, *P* = 0.09.], mainly due to higher values in the rats belonging to the SE models.

### Corticosterone Levels

**Figure 4:**
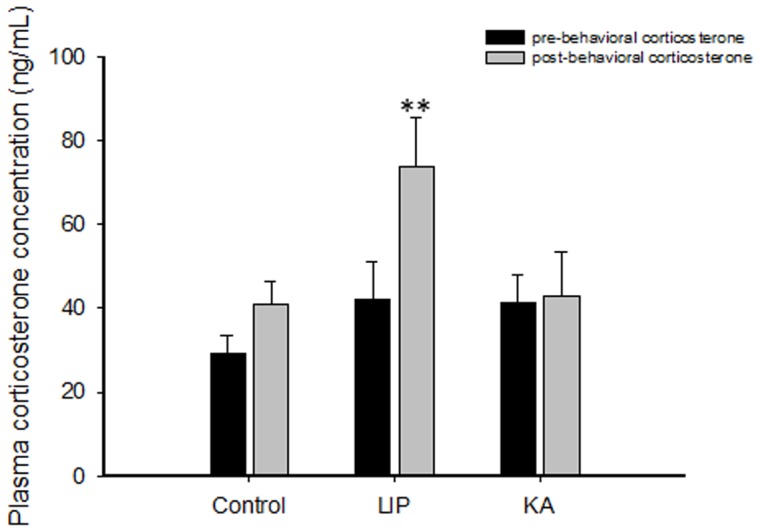
Plasma corticosterone levels assessed before (pre-behavioral phase) and after (post-behavioral phase) the behavioral tests. The results are presented as the mean ± SEM. ** P<0.01 vs. the corresponding pre-behavioral data from the same group. For this analysis, strains were pooled. Abbreviations: LIP: lithium-pilocarpine; KA: kainic acid.

The analysis of plasma corticosterone concentrations showed a main effect for the time at which samples were taken (pre- and post-behavioral) as well as an interaction between sampling and group [F(1, 26) = 8.43, *P*<0.01 and F(2, 26) = 7.41, *P*<0.01, respectively] but did not show a main effect for the group [F(2, 26) = 1.01, *P* = 0.38]. Subsequent Bonferroni-corrected pair-wise comparisons between each pair of samples for each group indicated that LIP-treated rats had a significantly higher post-behavioral corticosterone concentration (t = -4.60, df = 7, *P*<0.01) than their initial corticosterone concentration (Fig. 4). Interestingly, KA-treated animals did not show differences between the two samples (t = 0.38, df = 13, *P* = 0.71), similar to control rats (t = -0.66, df = 7, *P* = 0.53).

### Correlations between Corticosterone Levels and Emotional Behavior

Altogether, our results showed differences between the LIP and KA SE models regarding the affective symptoms and the functional state of the HPA axis. To establish a connection between alterations in the HPA axis and emotional behavior, we carried out correlation analyses between the main measures of affective behavior in the behavioral tests performed and between those measures and corticosterone levels.

**Figure 5:**
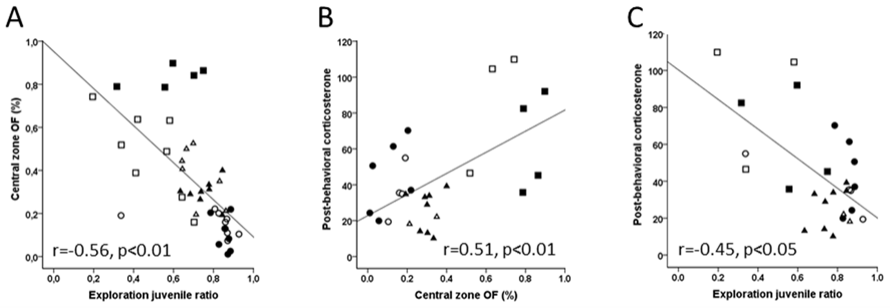
Statistical correlations (including all animals) between post-behavioral corticosterone levels and behavioral indices obtained in the social interaction and open field tasks. (A) Correlation between behavioral parameters in the open field and social interaction test. Symbol code: (•) control Wistar, (○) control Sprague-Dawley, (▪) lithium-pilocarpine Wistar, (□) lithium-pilocarpine Sprague-Dawley, (▴) kainate Wistar and (Δ) kainate Sprague-Dawley. (B) Correlation between the exploration in the inner zone in the open field and the post-behavioral corticosterone levels. (C) Correlation between the juvenile exploration ratio in the social interaction test and post-behavioral corticosterone level.

First, we evaluated whether there was correlation between anxiety- and depression-like behaviors. We found a negative correlation (r = -0.565, *P*<0.01) between the anxiety-like measurements from the open-field (OF) and behaviors in the social interaction test (Fig. 5A). In addition, we found a positive correlation between the time spent in the central area of the OF and the post-behavioral corticosterone level (r = 0.508, *P*<0.01; Fig. 5B), indicating that the greater the increase in the corticosterone levels (i.e., hyperactivity of the HPA axis), the lower the anxiety (i.e., the more time spent in the central area in the OF). Again, we found a negative correlation (r = -0.452, *P*<0.05; Fig. 5C) between post-behavioral corticosterone concentration and performance in the social interaction test, suggesting that the functional activation of the HPA axis is negatively related to the motivation for social interaction.

## Discussion

In this study, we investigated the effects of two SE models of TLE (LIP and KA) in two rat strains (Wistar and Sprague-Dawley) using tests for anxiety- and depression-like behaviors as well as measurements of plasma corticosterone responses. Our results showed two main findings. First, there are emotional differences between LIP- and KA-treated models. LIP-treated animals, irrespective of strain, exhibited alterations in anxiety and depression-like behaviors. However, KA-treated rats presented significantly less alteration than LIP-treated animals, and importantly, displaying only mild changes in social motivation and anxiety-like behaviors. Furthermore, no differences were observed between KA-treated animals and the control group in either anhedonic behavior or basal corticosterone levels. Second, LIP-, but not KA-, treated animals showed an increase in plasma corticosterone levels in post-behavioral measurements, indicating a hyperactivity of the HPA axis in this model of TLE.

Although anhedonia is considered a cardinal symptom of depression [25], we found no change in KA-treated rats and increased saccharin consumption in LIP-treated animals, irrespective of the strain. Despite clear epidemiological links between depression and epilepsy, there is limited experimental evidence from animal research to support a shared pathology. Reported findings using tests for anhedonia are conflicting, indicating no changes [26], [27], loss of taste preference [28] or even changes depending of the circadian rhythms [29]. Notably, in our study, the increase in saccharin consumption in LIP-treated rats was not associated with changes in water consumption or with increased locomotion, further suggesting that the effect was not due to changes in physiological processes that might interfere with ingestion or alter motor behavior. Previous studies also dismiss the notion of spontaneous seizure activity as a determining factor in depression-like behaviors, as no correlation between depression-like behaviors (i.e., anhedonia and despair measures) and seizure frequency was previously reported [28], [30].

Our data indicate a reduction in the motivation to explore a juvenile conspecific in both TLE models. This impairment is particularly pronounced in LIP-treated rats, while KA-treated rats show only a mild reduction in their social preference. The different exploratory behavior observed cannot be explained by altered locomotor behavior in the test. Because reduced social exploration in rodents is interpreted as a depressive-like phenotype [31], these data suggest that SE models lead to behavioral alterations that are congruent with depression-like symptoms. Moreover, differences in performance observed between LIP- and KA-treated rats suggest that differences in the extent of brain lesions [11] or in the anatomical organization of the temporal lobe caused by each of these two TLE models may underlie the differences in social behaviors [32].

We found clear differences in the time spent in the central zone of the open field, indicative of differences in anxiety-like behaviors between the SE models rat strains. Although KA-treated rats showed a mild reduction in anxiety levels, LIP-treated rats expressed very low levels of anxiety-like behavior. These data are in agreement with our previous observations using the elevated plus maze [11]. Anxiety-like behaviors reported in experimental models of epilepsy studies are contradictory, with some studies reporting increased anxiety levels [5], and others that accord with our results by indicating decreased anxiety levels [6], [33]. Previously [6], it has been hypothesized that these types of behavioral alterations might reflect disinhibited behavior. Ventral lesions of the hippocampus can alter unconditioned fear responses to threatening situations. Our previous study [11], in accordance with other reports [17], showed that TLE models induce extensive lesions of the ventral and dorsal hippocampus; particularly in LIP-treated animals, large lesions are also observed in the amygdala. Differences in the extent of the amygdala lesion might be responsible for variations in the assessment of threatening situations. This hypothesis is in agreement with our previous findings [11], which indicated that extension of brain damage, mostly affecting the lateral and basolateral amygdala, was correlated with another anxiety-related behavior test (the time spent in the open arms of an elevated plus maze). Consequently, these observations suggest that differences in the emotional reactivity might be more related with the underlying brain lesions than with the epileptic condition *per se*.

We also observed subtle strain differences in the magnitude of the anxiety effects, in line with previous data indicating that the strain factor interferes with the expression of epileptic phenotypes and behavioral performance [29], [34], [35]. Genetic heterogeneity among various rodent strains has been linked with their different response to epileptogenic inducers [36]–[38]. Several studies have shown evidence indicating that genetic heterogeneity may account for the observed discrepancies in measures of seizure-induced neuronal damage and behavioral differences [34], [39], [40].

Our results are in agreement with previous evidence showing hyperactivity of the stress hormone corticosterone in animal models of epilepsy, as evaluated during seizures or either post-ictal or interictal activity [41]–[44]. Interestingly, Mazarati et al. [44] showed that experimental TLE (accompanied by interictal HPA dysregulation) is correlated with increased depressive behaviors in the forced swimming test. It should also be noted that most of the behavioral tests typically used to evaluate depressive symptoms are also potentially stressful for animals (e.g., forced swimming test, social interaction test) and might produce a stress-induced hyper-reactivity of the HPA axis, including an increase in glucocorticoids [45]. Given that only LIP-treated rats presented alterations in corticosterone levels, it is tempting to suggest that their behavior in anhedonic and sociability tests might be affected by dysregulation of HPA activity and their response to stress.

In humans [46], [47] and animals [44], HPA hyperactivity was shown to be independent of seizure frequency, suggesting that chronic HPA dysfunction is associated with the epileptic condition. In a previous report, we did not find apparent differences in the seizure severity between SE models and strains [11]. Instead, we propose that alterations in the HPA axis are not part of the epileptic condition but, rather, are associated with the extension of the brain lesions induced by SE [11], [16], [32]. The minor impairment of emotional capabilities in KA- versus LIP-treated animals suggests that rats submitted to SE do not necessarily develop the same severity of affective disorders or HPA dysfunction. Moreover, our previous report [11] showed that there are important differences between models regarding the severity of brain lesions produced by SE; thus, pilocarpine and kainate models already exhibit distinct lesional patterns 24 h after SE [16]. In pilocarpine-injected rats, substantial multifocal damage is produced, affecting parahippocampal cortical regions, the thalamus, the endopiriform cortex and the amygdaloid nuclei [14]–[16]. In contrast, the kainate SE model induces less acute damage, with brain areas affected at a lower intensity than in the pilocarpine SE model [16]. There are important anatomical and functional connections between the hippocampus, amygdala and the HPA axis [48], [49], and a link between dysregulation of HPA axis and depression and anxiety disorders is also well known [50]. In agreement with previous data [51], we found reduced levels of anxiety-like behavior in the open field in models with clear damage of the amygdala but not in animals with intact amygdala nuclei, further implicating a functional separation between the TLE condition and anxiety-like disorders. In addition, our results suggest that this functional separation can impact depressive symptoms, which are manifested as a decrease in social motivation exhibited by LIP-treated animals. In accordance with these data, we argue that epileptic animals with severe damage to the amygdala and hippocampus are more likely to exhibit a dysregulation of the HPA axis and alterations in social interaction tests. In support of this idea, we found high correlations between the post-behavioral increase in plasma corticosterone and emotional behaviors.

In conclusion, we suggest that altered emotional behaviors are not necessarily part of the epileptic condition in experimental models of TLE; instead, they are likely to reflect a shift in anxiety levels related to a dysregulation of the HPA axis caused by model-dependent lesions.
